# Intraoperative assessment of bone viability through improved analysis and visualization of dynamic contrast-enhanced fluorescence imaging: technique report

**DOI:** 10.1097/OI9.0000000000000222

**Published:** 2022-11-30

**Authors:** Jonathan Thomas Elliott, Shudong Jiang, Eric R. Henderson, Gerard P. Slobogean, Nathan N. O'Hara, Cao Xu, Jing Xin, Xinyue Han, Melanie L. Christian, Ida Leah Gitajn

**Affiliations:** a Department of Orthopaedics and Sports Medicine, Dartmouth-Hitchcock Clinic, Lebanon, NH,; b Thayer School of Engineering at Dartmouth, Hanover, NH, and; c University of Maryland School of Medicine, R Adams Cowley Shock Trauma Center, Department of Orthopaedics, Baltimore, MD.

**Keywords:** debridement, fluorescence-guided surgery, fracture fixation, open fracture, surgical wound infection, trauma

## Abstract

Bone devitalization is believed to be a critical determinant of complications such as infection or nonunion. However, intraoperative assessment of bone devitalization, particularly in open fractures and infections, remains highly subjective resulting in variation in treatment. Optical imaging tools, particularly dynamic contrast-enhanced fluorescence imaging, can provide real-time, intraoperative assessment of bone and soft tissue perfusion, which informs the tissues' ability to heal and fight infection. We describe a novel technique to apply indocyanine green–based fluorescence imaging, using a device that is frequently used in the operating room to assess skin or flap perfusion in plastic surgery, to assess bone and deep tissue perfusion in three pertinent cases: (1) a chronic infection/nonunion after a Gustilo type 3A tibia fracture (patient 1), (2) an acute Gustilo type 3C tibia open fracture with extensive degloving/soft tissue stripping (patient 2), and (3) an atrophic nonunion of the humerus (patient 3). In all three cases, fluorescence imaging (both time-specific fluorescence and maximum fluorescence) and derived kinetic maps of time-to-peak, ingress slope, and egress slope demonstrated clear spatial variation in perfusion that corresponded to the patient pathogenesis. The impact of this information on patient outcome will need to be evaluated in future clinical trials; however, these cases demonstrate in principle that optical imaging information has the potential to inform surgical practice, reduce the variation in treatment, and improve outcomes observed in these challenging patients.

## 1. Introduction

Dysvascular tissues have limited potential to regenerate, heal, or fight infection due to insufficient delivery of osteoprogenitor cells, growth factors, inflammatory cells, endogenous immune cells, and antibiotics. Because of this, the cornerstone of treatment of contaminated open fracture, established infection, and selected atrophic nonunion is to achieve a thorough debridement of poorly perfused or nonviable bone.[1–3] To date, the eyes and hands of the surgeon remain the dominant “imaging modality” used to make decisions regarding health of both soft tissue and bone. However, palpation and visual inspection are not always sufficient for discriminating between healthy and nonviable tissue, and inaccurate assessment can have important downstream consequences. In the context of open fracture, infection, and some nonunions, more extensive debridement may minimize the risk of further complication^[2,3]^; however, this comes at the cost of increasingly complex reconstructive procedures to fill bony defects.^[4–6]^ The subjective nature of this assessment likely results in substantial variation in the extent of debridement, particularly when comparing more-experienced and less-experienced surgeons, which may place patients at unnecessarily high risk of complications. Optical imaging tools are being increasingly applied to a wide array of surgical fields using various targeted and untargeted fluorescent probes to enhance contrast and understand tissue kinetics. In surgical oncology, glioma resections are now routinely guided by 5-aminolevulinic acid visualization, which has improved median survival in high-grade glioma.^[7]^ In bowel anastomoses, indocyanine green fluorescence imaging can provide surgeons with intraoperative data beyond what can be seen and palpated, differentiating between perfused and dysvascular bowel.^[8,9]^ Recently, it has been observed that certain antibiotics (eg, tetracycline and doxycycline) that are incorporated into bone formation and regeneration processes can be used in humans to identify necrotic bone regions in bisphosphonate-related osteonecrosis of the jaw. In a study of 10 patients, fluorescence imaging was superior to more clinically subjective indicators such as bone bleeding in patients with bisphosphonate-related osteonecrosis of the jaw.^[10]^ There is enormous untapped potential to apply these tools to improve care of musculoskeletal trauma, but unlocking this depends on (1) availability of fluorescence imaging systems, (2) advancements in quantitative analysis, and (3) intraoperative visualization of this information. To improve the objective assessment of bone perfusion, we present our initial, first-in-human experience applying indocyanine green (ICG)–based dynamic contrast-enhanced fluorescence imaging using data processing and analytic strategies customized for bone imaging in three patients to assess bone vascularity in high-energy open fracture, post-traumatic infection, and chronic atrophic nonunion.

## 2. Surgical Technique

### 2.1 Intraoperative Dynamic Contrast-Enhanced Fluorescence Imaging

**Figure 1.**
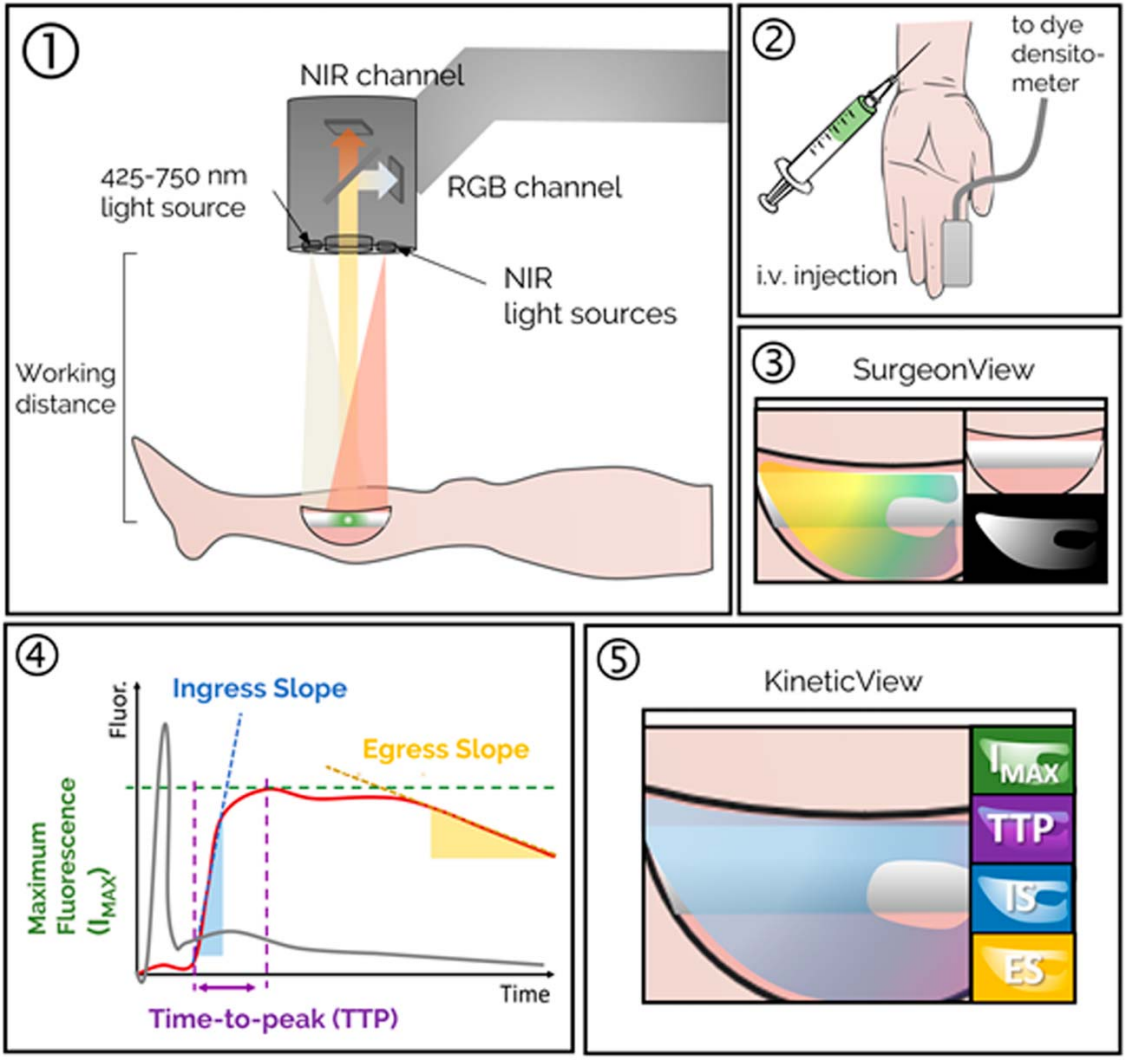
Step 1: Fluorescence imaging system is positioned over the surgical field, and fluorescence video acquisition is started. Step 2: A dye densitometer finger probe acquires the time-dependent arterial dye concentration (called arterial input function). Dye is injected intravenously. Step 3: Real-time fluorescence video, including overlay onto the color image of the surgical field, is displayed on the “SurgeonView.” Step 4: Collection of video rate recorded data allows for analysis of the inflow/outflow kinetics, which can be assessed using several parameters including maximum fluorescence intensity (I_max_), time-to-peak (TTP), ingress or inflow slope (IS), and egress or outflow slope (ES). These parameters are computed for the time–concentration curve of each pixel (solid red line), corrected to account for differences in arterial input function (solid gray line), to produce kinetic maps. Step 5: The kinetic maps can then be displayed on a secondary monitor “KineticView” so that both the real-time SurgeonView information and the computed KineticView information are available intraoperatively.

In the operating room, the surgical site was exposed and self-retainers were placed into the wound. A sterile drape was then placed over the SPY Elite camera. The SPY Elite system was positioned at a 300-mm working distance from the surgical field. A white light image was obtained, and then, video rate fluorescence images were recorded every 0.3 seconds for 4.5 minutes (Fig. 1). A pulse dye densitometer^[11,12]^ (similar to a pulse oximetry probe) was placed on the finger at the start of the procedure to collect a patient-specific arterial input function, which permits measurement of ICG injection parameters. This measurement allows post hoc correction of variation in injection parameters. Video rate recorded images were obtained for 20 seconds before injection of ICG contrast to establish a baseline value. After 20 seconds, baseline measurements were obtained, 0.1 mg/kg of ICG was administered intravenously, and the video rate recording was completed for a total of 4 minutes. White light images of the surgical site were also obtained.

In the three patients presented, the surgical debridement proceeded per standard of care without using ICG-based fluorescence imaging.

### 2.2 Enhanced Kinetic Analysis and Visualization of Dynamic Contrast-Enhanced Images

After acquisition of 1024 frames (maximum with SPY Elite), DICOM images were saved and loaded into in-house developed software. Coregistration between the RGB and the fluorescence stack is performed before the single time-point fluorescence intensity images are displayed in the “SurgeonView” panel. Simple curve analysis is applied to the fluorescence time series, which were first corrected by the arterial input function to account for any variations in how the ICG dye is injected and for allometric variation because the dye is dosed according to weight.^[12]^ Kinetic parameter maps such as maximum intensity (I_max_), time-to-peak (TTP), ingress slope (IS), and egress slope (ES) are shown in a more detailed panel termed “enhanced DCE view” (Figs. 3 and 5). The research assistant or clinical personnel can click the thumbnails on the left of the panel to select that map as the large “fused” main image. By clicking the large image, points of interest (red, blue, and yellow dots) can be selected, and the time–concentration curve for those locations updates in the lower-middle panel. In this way, the surgeon can quickly identify regions of hypoperfusion and venous congestion.

## 3. Clinical Experience

After IRB approval by the Dartmouth-Hitchcock Human Research Protection Program (Study No. 31575 PI: I. L. Gitajn), we report on the results of ICG-based dynamic contrast-enhanced fluorescence imaging (DCE-FI)^[13]^ in the operating room for 3 patients: patient 1 with a chronic infected nonunion after an open Gustilo type 3A open tibia fracture, patient 2 with an acute Gustilo type 3C open distal tibial fracture, and patient 3 with a chronic atrophic humeral nonunion. The study was approved by the local IRB in accordance with the declarations of the World Medical Association (www.wma.net). All patients provided written informed consent before the procedure.

### 3.1 Infection Case

**Figure 2.**
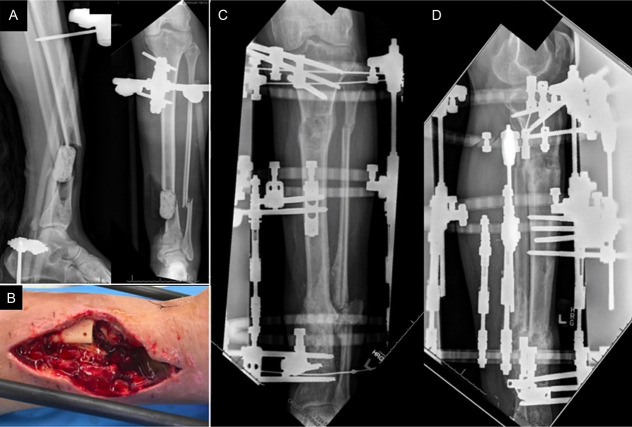
Radiograph (A) and clinical image (B) after initial debridement and removal of intramedullary nail. Radiographs after revision debridement followed by distraction osteogenesis/bone transport (C, D).

**Figure 3.**
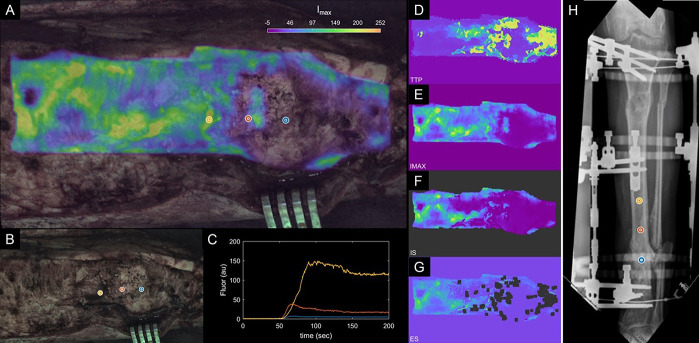
A, “Enhanced DCE view” of patient 1 data showing maximum intensity heat map overlaid on the white light image. B, White light intraoperative image. C, Kinetic ICG inflow/outflow curves at regions of interest identified with blue, red, and yellow dots. D–G, Kinetic heat maps representing ICG time-to-peak (D), maximum ICG intensity (E), ICG inflow slope (F), and ICG outflow slope (G). H, AP radiographs with regions of interest (blue, red, yellow). ICG, indocyanine green.

Patient 1 is a 57-year-old healthy active man involved in a motorcycle collision 2 years prior resulting in an open Gustilo type 3A distal third tibia fracture, which was complicated by acute infection after routine debridement and intramedullary fixation (Fig. 2A and B). The infection was treated with removal of the intramedullary device and extensive debridement of poorly perfused bone with an anterolateral thigh flap to cover the dehisced wound. The patient initially elected to proceed with Masquelet bone grafting, which failed to unite after two bone grafting procedures; he was subsequently managed through bone resection with acute shortening, iliac crest bone grafting with compression of the fracture site, and distraction osteogenesis proximally to reestablish limb length (Fig. 2C and D). Because the radiographic and computed tomography (CT) imagings were equivocal regarding bony union at the docking site, several struts were removed from the external fixator to “road-test” the docking site, and within 2 weeks, he experienced increasing pain and progressive deformity at that site confirmed the persistent nonunion. Rather than undergoing additional bony resection, he elected to proceed with a transtibial amputation. At the time of amputation, his tibia was exposed and dynamic contrast-enhanced fluorescence imaging was performed. White light images (Fig. 3B) were obtained in concert with fluorescence imaging. The surgeon identified the nonunited region as bone with presumed limited viability based on clinical intraoperative findings. This was reflected by the ICG-based perfusion imaging (Fig. 3). Region of interest (ROI) 1 was at the docking site/nonunion site (blue), ROI 2 was 2 cm proximal to the docking site (red), and ROI 3 (yellow) was further proximal by 2 cm increments (Fig. 3). Fluorescence intensity in the farthest from the fracture site was 195 RFUs, whereas maximum fluorescence intensity at the docking site was 8 RFUs (24× lower). Kinetic inflow/outflow curves of the ICG into and out of bone demonstrate an increase in the maximum fluorescence intensity and a change in the shape of the kinetic curve as bone becomes healthier more proximally (Fig 3A and 3C). All perfusion variables (maximum intensity and kinetic curve–related variables) demonstrated improved vascularity at the more proximal sites (Table 1). At 12-month follow-up, patient 1 was doing well, mobilizing independently with the use of a below-the-knee prosthesis.

**TABLE 1:** Kinetic Map Parameters for Regions of Bone Defined as “Injury” by the Surgeon During the Time of Surgery, and Adjacent “Background” Regions of Bone, for All Three Patients, Including Predebridement and Postdebridement Values for Patient 3

Parameter	Patient 1		Patient 2		Patient 3 (predebridement)		Patient 3 (postdebridement)	
	Injury	Background	Injury	Background	Injury	Background	Injury	Background
Max fluorescence (RFUs)	111 ± 21	156 ± 15	104 ± 21	152 ± 13	26 ± 6	141 ± 75	102 ± 36	105 ± 39
TTP (s)	149 ± 39	103 ± 25	184 ± 22	120 ± 40	82 ± 54	133 ± 71	161 ± 42	162 ± 63
Ingress slope (RFU/s)	17.1 ± 3.0	26.7 ± 3.4	15.0 ± 0.6	24.6 ± 6.8	1.5 ± 1.8	1.8 ± 3.0	0.6 ± 0.6	0.9 ± 1.5
Egress slope (RFU/s)	1.8 ± 0.6	2.9 ± 0.5	2.1 ± 0.6	2.7 ± 0.4	2.7 ± 0.6	2.1 ± 1.5	1.2 ± 1.5	0.6 ± 1.2

Values are represented as mean ± SD.

SD, standard deviation; TTP, time-to-peak.

### 3.2 Open Fracture Case

**Figure 4.**
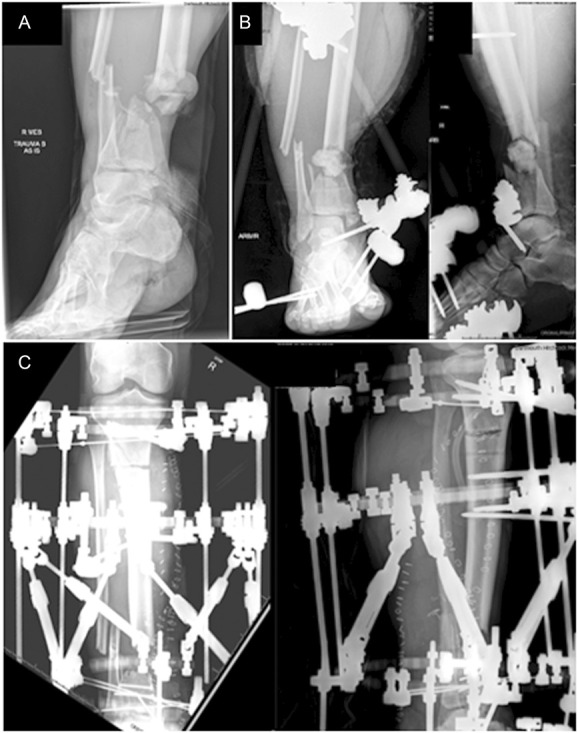
Injury radiograph (A), initial postdebridement radiographs (B), and radiographs after frame placement with proximal tibial osteotomy for distraction osteogenesis/bone transport (C).

Patient 2 is a 50-year-old man who was involved in a motorcycle collision resulting in a Gustilo Type 3C open distal tibia fracture with extensive degloving and periosteal stripping. He went to the operating room urgently for vascular reconstruction and irrigation and debridement of the open contaminated fracture (Fig. 4). At his second debridement procedure, dynamic contrast-enhanced fluorescence imaging was obtained of his open fracture site to assess the viability of the comminuted and degloved distal tibia. White light images were obtained in concert with fluorescence imaging. His bone defect was definitively treated with distraction osteogenesis/bone transport in a circular external fixator (Fig. 4).

**Figure 5.**
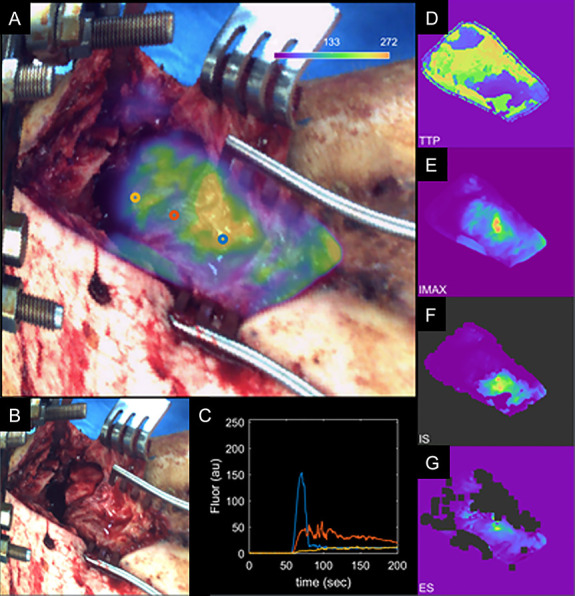
A, “Enhanced DCE view” of patient 2 showing maximum intensity heat map overlaid on the white light image. B, White light intraoperative image. C, Kinetic ICG inflow/outflow curves at regions of interest identified with the yellow, red, and blue dots. D–G, Kinetic heat maps representing ICG time-to-peak (D), maximum ICG intensity (E), ICG inflow slope (F), and ICG outflow slope (G). ICG, indocyanine green.

Based on the severity of the injury in association with extensive periosteal stripping, the fracture region was believed to be relatively dysvascular based on clinical assessment. This was reflected in the ICG-based perfusion imaging. After debridement was completed, ICG-based fluorescence imaging was performed (Fig. 5). Figure 5 shows maximum fluorescence intensity overlaid on the white light image in Figure 5A, demonstrating that the maximum perfusion improves more proximally, away from the fracture (yellow ROI at the fracture, blue ROI being more proximal). This is also reflected in the kinetic curves demonstrated in Figure 5C and the kinetic heat maps in Figure 5D–5G, which demonstrate the other kinetic curve–related variables (time-to-peak, maximum ICG intensity, IS, and ES). At 12-month follow-up, patient 2 was doing well. He completed bone transport with fracture union at the site of the segmental defect. He has a plantigrade foot and mobilizes with the use of an AFO brace for his persistent foot drop.

### 3.3 Nonunion Case

**Figure 6.**
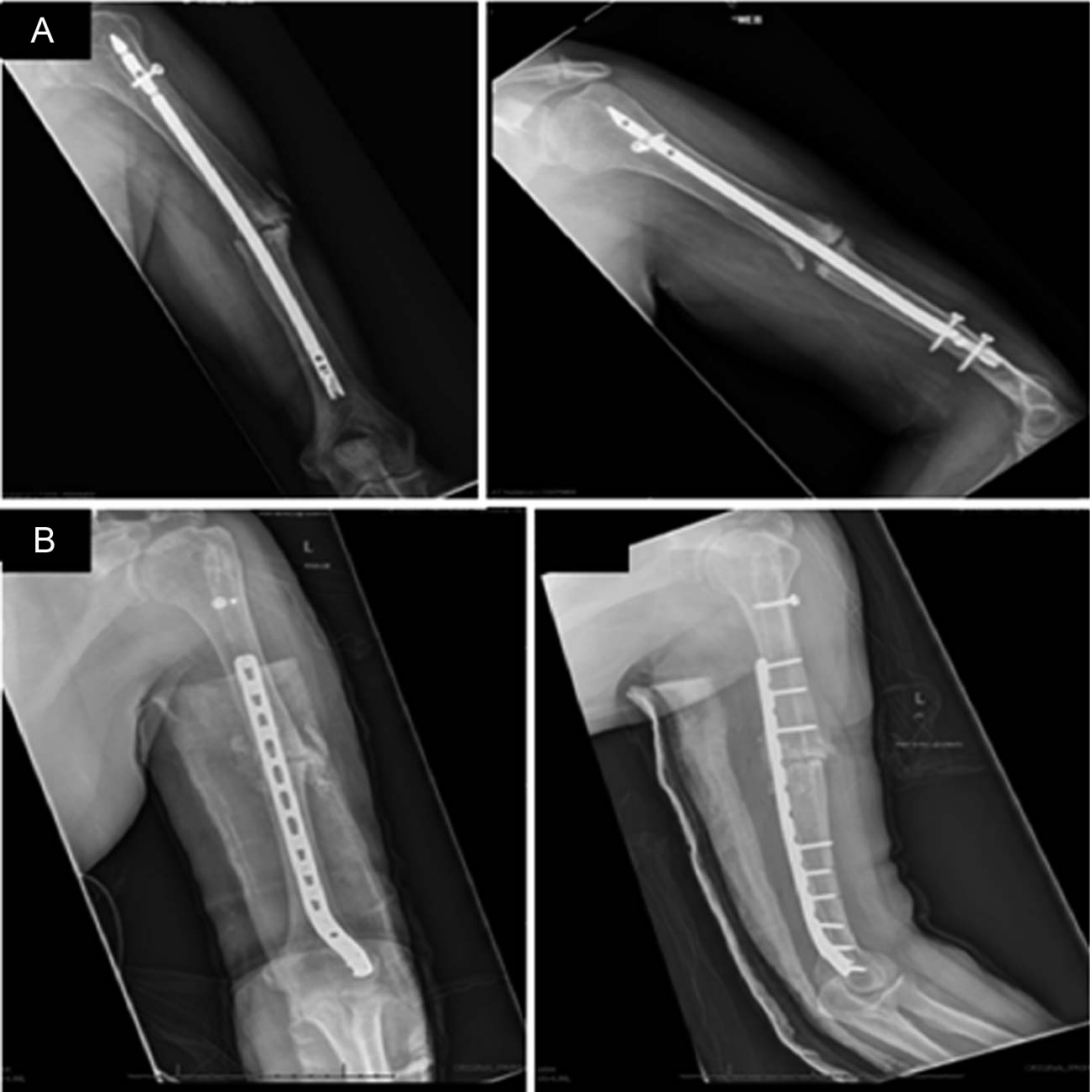
Preoperative radiographs of the atrophic humeral nonunion (A) and postoperative radiographs after compression plating, debridement with acute shortening, and iliac crest bone graft (B).

Patient 3 is a 61-year-old man with an atrophic nonunion after intramedullary fixation of his open humeral fracture approximately 20 years ago. He presented after having sustained a ground-level fall resulting in a broken interlocking screw, new worsening pain, and gross motion at the nonunion site (Fig. 6). All incisions were benign-appearing, and all nonunion laboratory tests, including inflammatory markers, were benign. He underwent removal of the retrograde humeral nail, debridement and acute shortening of his humeral shaft, and compression plating with iliac crest autograft using a posterolateral approach to the humerus.

**Figure 7.**
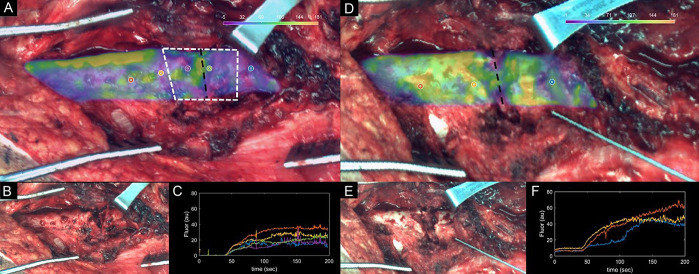
(A) Predibridement and (D) postdebridement kinetic maps of patient 3, showing I_max_ kinetic maps overlaid on (B and E) white light intraoperative images. Improvements in kinetic curves can be observed from (C) predebridement to (F) postdebridement, suggesting procedure was effective.

Based on the clinical and radiographic assessment, the nonunion region was considered atrophic and relatively dysvascular. This was reflected in the ICG-based perfusion imaging. ICG fluorescence imaging was obtained before and after bone excision/acute humeral shortening. Before acute shortening, there is dysvascular bone both proximal and distal to the nonunion as reflected in the maximum fluorescence intensity maps (Fig. 7A). After acute shortening, the dysvascular bone proximal to the nonunion had been resected (Fig. 7D). Similar to the cases above, bone perfusion seems to improve in RIOs sequentially further away from the nonunion site.

**Figure 8.**
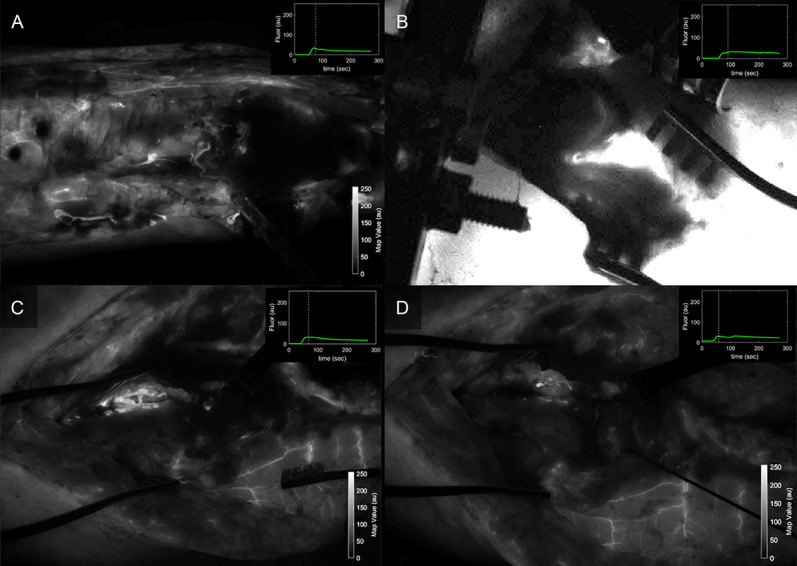
Fluorescence intensity maps acquired during the early wash-out period after ICG injection for (A) patient 1, (B) patient 2, and patient 3 (C) predebridement and (D) postdebridement, as seen with the SPY Elite standard visualization available in clinical units. ICG, indocyanine green.

Parametric map values obtained from regions of interest corresponding to the injury site and the normal background site in each patient are summarized in Table 1. These values highlight what can be observed visually in Figures 3, 5, and 7: The site of injury is consistently associated with an increase in time-to-peak and a decrease in maximum fluorescence, ingress slope, and egress slope. For comparison between these curve-related parameters and the standard images acquired with the clinical device, Figure 8 shows the images as visualized on the SPY Elite system after ICG injections. At 9-month follow-up, patient 3 did go on to a successful bony union, visible starting at his 3-month postoperative radiographs with progressive improvement to complete union.

## 4. Discussion

Decision-making about the extent of bone and soft tissue debridement in the setting of complex orthopaedic surgical conditions such as high-energy open traumatic wounds, infections, and select atrophic nonunions remain routine and challenging intraoperative dilemmas. ICG-based dynamic contrast-enhanced fluorescence imaging has been used for more than 50 years in intraoperative settings to assess vascular perfusion of a wide variety of tissues. In this study, we have translated preclinical ICG fluorescence-based perfusion imaging into human patients. Our preclinical data suggest that ICG-based DCE-FI can effectively and quantitatively assess bone viability in real time.^[14]^ In the infected nonunion case (patient 1), there were clear trends demonstrating extremely poor vascular perfusion to the infected nonunion site with improving perfusion at each more proximal ROI, suggesting that the patient's failure to heal or clear the infection may be due to an inadequate surgical debridement/bony resection. In the open fracture case (patient 2), there was vascular perfusion at the fracture site, which did improve more proximally/farther away from the fracture. However, the fracture site did ultimately go on to union. In the nonunion case (patient 3) before debridement/acute shortening, perfusion of bone both proximal and distal to the nonunion was clearly poorer than more proximal bone. After bone resection, the poorly perfused bone proximal to the nonunion was resected; however, the bone distal to the nonunion was improved but still seemed deficient compared with surrounding regions, suggesting that the resection may have been inadequate to remove all poorly perfused bone. The differences between injured or clinically suspicious regions and normal regions for each of the kinetic maps and for all three patients are summarized in Table 1. In all three patients, kinetic parameters show marked differences between injured and noninjured regions. While more work is needed before these parameters can be used in a diagnostic sense, this early translational work suggests that intraoperative ICG-based dynamic contrast-enhanced fluorescence imaging can provide clinically useful information about vascular perfusion of bone.

Bone blood flow is critical to many processes including growth, bone remodeling, maintenance of bony strength, fracture healing, ingrowth after joint replacement, prevention of infection, and treatment of infection.^[15,16]^ Changes in bone blood flow is implicated in morbidity associated with tobacco smoke, osteoporosis, osteoarthritis, and diabetes and in bone infection.^[17–26]^ In the setting of traumatic injury, damaged blood flow to bone and soft tissue is believed to hinder the delivery of antibiotics, endogenous immune cells as well as growth factors, and other building blocks for healing traumatized tissues and preventing infection. In the setting of open fracture, poorly perfused bone then becomes a nidus for biofilm formation from environmental contamination.

ICG-based dynamic contrast-enhanced fluorescence imaging is a powerful imaging method which can be used to better understand several components of vascular perfusion reflected in different kinetic curve–related variables such as rate of ingress (wash-in slope), rate of egress (wash-out slope), time needed to reach peak (blood perfusion), and volume of blood getting to tissues (maximum intensity) (Fig. 1). Furthermore, complex modeling techniques can be applied to these kinetic data to differentiate endosteal from periosteal blood supply, as we have demonstrated in our previous work.^[27]^ This has profound clinically oriented applications in the context of traumatic injury in which fracture is believed to disrupt endosteal blood flow, and soft tissue stripping is believed to disrupt periosteal blood flow. This has the potential to provide surgeons with a nuanced view of vascular perfusion parameters in surgical tissues in real time in the operating room. However, more work is needed to define which curve-related variables or vascular perfusion parameters are better predictors of outcomes of interest (such as complication or infection) and where thresholds may be for those parameters. Furthermore, histopathology may be used in future research to validate the perfusion-based findings.

More conventional imaging modalities have been evaluated in this context in a relatively limited way. In a small eight-patient case series, positron emission tomography/CT (PET/CT) was applied to patients with chronic osteomyelitis to inform preoperative planning of debridement.^[1]^ Two studies have demonstrated that after nonunion surgery perfusion assessment using dynamic contrast-enhanced magnetic resonance imaging is associated with treatment failure/persistent nonunion,^[28,29]^ which highlights the importance of blood flow assessment in this patient population. However, there are critical shortcomings associated with both PET/CT and MR techniques for surgical guidance. PET/CT is time intensive and has substantial issues with poor resolution.^[30,31]^ High spatial resolution is needed to accurately detect regions of poor vascularity surrounded by regions of higher vascularity. DCE-MRI techniques are limited in orthopaedic patients because of metal artifact/signal dropout in association with metal implants.^[14]^ Furthermore, there is substantial cost associated with PET/CT and DCE-MRI, and neither of these imaging modalities are currently feasible for use in the operating room in real time. Owing to analogous goals regarding improving margins in oncologic resections, surgical oncology is increasingly turning to intraoperative real-time fluorescence-guided surgical techniques,^[32]^ and we believe that there is enormous potential in orthopaedic surgical applications as well.

In the patients presented here, we demonstrated that ICG-based DCE-FI demonstrates considerable promise in providing surgeons with an objective measure of bone and soft tissue viability, which has the potential to substantially advance management of severe traumatic injury. We believe that providing surgeons with quantitative objective data on vascular perfusion of surgical bone and soft tissues will result in improved ability to assess and perform effective debridement, which may result in decreased variability in treatment and possibly reduced the rate of complication. The use of current commercial fluorescence imaging devices, such as the SPY presented here, requires minimal training to operate, and the per patient cost of contrast agent is less than $100. Indocyanine green has a very strong safety profile and has been used for more than 50 years to determine hepatic function and cardiac output. The use of DCE-FI may be particularly useful for guiding new and inexperienced orthopaedic surgeons or providing care in more remote and austere settings. However, more research is needed to evaluate those questions and to optimize fluorescence imaging methods for these purposes. Based off these results, a larger clinical trial is underway.
